# Hepatic Failure Related to Itraconazole Use Successfully Treated by Corticosteroids

**DOI:** 10.5812/kowsar.1735143X.755

**Published:** 2011-10-01

**Authors:** Horng-Yuan Lou, Chia-Lang Fang, Sheng-Uei Fang, Cheng Tiong, Yang-Chih Cheng, Chun-Chao Chang

**Affiliations:** 1 Division of Gastroenterology and Hepatology, Department of Internal Medicine, Taipei Medical University Hospital, Taipei, Taiwan; 2 Department of Pathology, Taipei Medical University Hospital, Taipei, Taiwan; 3 Department of Internal Medicine, School of Medicine, College of Medicine, Taipei Medical University, Taipei, Taiwan

**Keywords:** Itraconazole, Adrenal Cortex Hormones, Liver Failure

## Abstract

**Background:**

Itraconazole is believed to carry a low risk of hepatic toxicity owing to its low affinity for the human P-450 enzyme. Therefore, hepatic failure caused by itraconazole is exceedingly rare.

**Objectives:**

We report the case of a 46-year-old woman who developed hepatic failure related to itraconazole that was administered for the treatment of onychomycosis. Her condition deteriorated after withdrawal of the drug, followed solely by supportive care initially.

**Case Report:**

Treatment with corticosteroids was started 10 days after her admission, and her condition gradually improved. Unfortunately, her condition worsened when the dosage of corticosteroids was abruptly decreased. Ultimately, her condition improved with appropriate adjustments of corticosteroid dosage.

**Discussion:**

We conclude that corticosteroid therapy may be effective for itraconazole-induced hepatitis, especially in those patients who do not respond to conservative treatment. Notably, any decrease in the dosage should be performed with caution. We also recommend that close monitoring of liver function is mandatory during the use of itraconazole.

## 1. Background

Itraconazole has been subjected to thorough clinical evaluation since 1983. It has been extensively used in the treatment of superficial mycoses including vaginal and oral candidiasis, dermatophytosis, and systemic mycoses [1]. The most frequently reported adverse events are gastrointestinal disturbance and skin reactions. The rate of abnormal results of liver function tests and severe hepatitis among itraconazole users has been estimated to be 3% and less than 2%, respectively. In most cases, patients recover spontaneously after discontinuation of itraconazole treatment and administration of supportive treatment. However, the liver function of patients receive long-term therapy should be closely monitored, because liver dysfunction in these patients has been reported to be fatal. Liver transplantation in such cases is reserved for those patients who show severe liver damage.

## 2. Objectives

Corticosteroids are widely used in the treatment of a variety of autoimmune diseases. Only a few case reports on the usage of corticosteroids for the treatment of druginduced hepatitis are available. In this study, we report the successful use of corticosteroids in the treatment of hepatic failure caused by itraconazole.

## 3. Case Report

**Figure 1:**
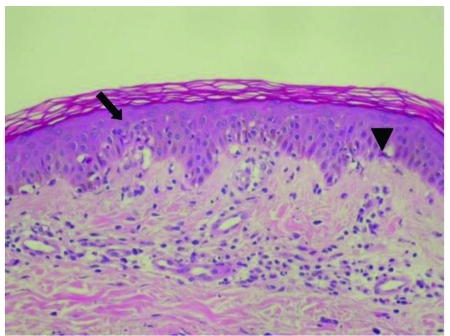
Epidermal Changes in Erythema Multiforme with Vacuolated Degeneration of Basal Cells (↓) and Scattered Individual Keratinocyte Necrosis (▼). Hematoxylin and Eosin, 200×

**Figure 2:**
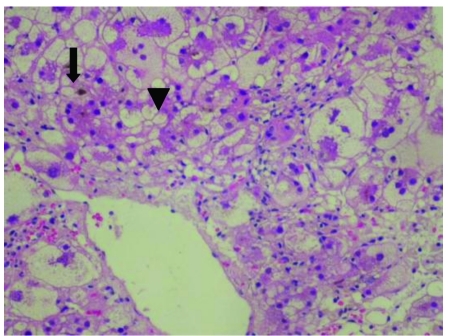
Degeneration of Hepatocytes Indicated by a Prominent, Centrilobular Zonal Ballooning ( ↓) with Cholestasis in the Bile Canaliculi ( ▼). Hematoxylin and Eosin, 200×

A 46-year-old woman with anorexia and jaundice was admitted to the Taipei Medical University Hospital. Her medical history was unremarkable, except for onychomycosis of both toes, which had been treated with itraconazole (200 mg daily) for 5 weeks. She gradually developed symptoms of weakness and nausea. Generalized pruritus developed 1 week before admission. On examination, she was found to have jaundice without abdominal  tenderness or hepatosplenomegaly. The initial laboratory data were as follows: alanine transaminase (AST), 2571 IU/L; aspartate transaminase (ALT), 3789 IU/L; total bilirubin, 4.4 mg/dL; albumin, 3.1 g/dL; prothrombin time, 13.57 s; and international normalized ratio (INR), 1.43. The test results for serum antinuclear antibody and anti-smooth muscle antibody were negative; moreover, a viral hepatitis survey that included tests for hepatitis A, B, C, and E yielded negative results. Five days after her admission, multiple erythematous papules appeared on her lower extremities. A skin biopsy showed prominent perivascular lymphocytes and occasionally eosinophil infiltrations. The epidermis showed focal spongiosis, basal layer vacuolization, leukocyte exocytosis, and keratinocyte necrosis (Figure 1). The pathological findings were compatible with a clinical diagnosis of erythema multiforme. Her condition deteriorated despite supportive treatment. She developed ascites 10 days after admission. The results of her liver chemistry tests were as follows: AST, 532 IU/L; ALT, 1298 IU/L; total bilirubin, 12.8 mg/ dL; albumin, 2.3 g/dL; prothrombin time, 18.35 s; and INR, 2.77. Intravenous methylprednisolone was initially administered at 40 mg every 8 hours, and the dose was gradually decreased to 20 mg every 12 hours over the course of 1 week. Her condition dramatically improved, and the ascites disappeared completely. The laboratory data were as follows: total bilirubin, 8.8 mg/dL; prothrombin time, 12.75 s; and INR, 1.24. Thereafter, 10 mg of oral prednisolone was administered every 12 hours to replace the 20 mg intravenous methylprednisolone. However, her condition deteriorated to some extent after this change, and her total bilirubin level reached 12.7 mg/dL. A liver biopsy detected cholestatic hepatitis with marked bile plugs that were accumulated in the canaliculi and surrounded  by hepatocytes in a rossetting pattern. Degeneration of hepatocytes indicated by a prominent, pericentral zonal ballooning was observed (Figure 2). Patchy necrosis of the hepatocytes was also noted. In the portal and periportal areas, lymphocyte and neutrophil infiltration was observed. The patient’s condition was consistent with  acute cytotoxic and cholestatic injuries caused by druginduced hepatotoxicity. Intravenous injection of 20 mg methylprednisolone was restarted and administered every 8 hours. This dosage was gradually decreased over the following 2 weeks. The patient’s condition stabilized after this adjustment. She was discharged 36 days after her admission. The laboratory data at the time of discharge were as follows: total bilirubin, 3.3 mg/dL; AST, 116 IU/L; and ALT, 186 IU/L. The levels of the parameters measured during the course of the disease are shown in Table. Oral prednisolone at 5 mg daily was continued for 1 week after her discharge from the hospital. After 2 weeks, her liver chemistry test results returned to normal.

**Table:** The Level of Various Laboratory Parameters at Different Time Point of the Disease Course

	**AST a ,(0-40 IU/L)**	**ALT a ,(0-40 IU/L)**	**Total Bilirubin,(0-1.4 mg/dL)**	**Albumin,(3.5-5.2 g/dL)**	**Prothrombin Time,(8.0-12.0 sec)**	**INR a ,(0.85-1.15)**
Initial	2571	3789	4.4	3.1	13.57	1.43
10 days after admission	532	1298	12.8	2.3	18.35	2.77
After methylprednisolone injection	188	641	8.8	2.9	12.75	1.24
Rapid switch to oralprednisolone	168	398	12.7	2.7	12.17	1.17
Before discharge	116	186	3.3	3.1	11.22	0.94

^a^ Abbreviations: ALT, alanine aminotransferase; AST, aspartate amoniteransferase; INR; international normalized ratio

## 4. Discussion

Itraconazole is an antifungal agent used in the treatment of deep and superficial fungal infections. It impairs the conversion of lanosterol to ergosterol by inhibiting the fungal cytochrome P450-dependent enzyme. The effect of itraconazole on the mammalian cytochrome P450 is lesser than that of other azoles, thereby suggesting a lesser effect on human steroidogenesis [2]. Therefore, hepatotoxic reactions caused by itraconazole have only rarely been reported. Adverse reactions to itraconazole include skin eruptions, gastrointestinal upset, thrombocytopenia, headache, dizziness, reversible edema of the extremities, gynecomastia, hypokalemia, and hypertriglyceridemia [3]. The level of serum transaminases increases in 1%–5% of patients who receive continuous therapy [4][5]. Symptomatic hepatitis rarely occurs and, when it does, recovery generally ensues with the cessation of medication [6]. The recovery period varies, as  reported in the literature. Talwalkar et al. reported a case of severe cholestasis related to itraconazole usage. The period of recovery from symptomatic hepatitis in the patient in that case was 5 months without any specific treatment [7]. In our study, we encountered a patient with severe hepatitis who developed hypoalbuminemia, ascites, jaundice, and coagulopathy. The case of this patient was different from most cases that have been reported by others, because her condition was protracted even after discontinuation of itraconazole therapy. Corticosteroids have well established efficacy in the treatment of autoimmune hepatitis. However, only a few reports on the successful treatment of drug-induced hepatitis by corticosteroids are available. These include reports on the treatment of drug-induced hepatitis by troglitazone [8] and ticlopidine [9]. Prednisolone had been used in the treatment of itraconazole-related hepatitis in 2 patients [10][11], but the liver damage in both the patients was not as severe as that in our patient. Our patient’s condition dramatically improved after corticosteroid treatment; however, she experienced a relapse when the dosage was abruptly reduced. Subsequently, she recovered after administration of adequate doses of corticosteroids. This observation implies that the response is highly corticosteroid dependent, a correlation that has never been reported in the literature. However, the mechanism underlying the treatment of itraconazole-related hepatitis by corticosteroids remains obscure and requires further investigation. Reports on the pathological features of itraconazole-induced hepatitis are still limited. Both cytolytic and cholestatic hepatitis have been reported to be caused by itraconazole administered at certain doses [12]. According to Adriaenssen’s [13] report of itraconazole- induced hepatitis in 3 patients, all the patients developed cholestatic liver injury with damage to the interlobular bile ducts. In addition, 2 of these patients showed ducopenia. None of them showed clinical hallmarks of an immunologic idiosyncrasy such as fever, rashes, or eosinophilia. The author concluded that itraconazoleinduced hepatitis is a metabolic rather than an immunoallergic idiosyncrasy. In contrast, histological evaluations by Tuccori [14] and Srebrnik [15] showed massive panlobular necrosis. Both of their patients showed a pattern of cytolytic liver injury without a skin reaction, and both required liver transplantation. In our patient, the predominant manifestation was a hepatocellular type of injury. She also developed fever and erythema multiforme. A similar case has not been reported in the literature. Whether pathological presentation is correlated with clinical management deserves further study.

The incidence of cutaneous reactions induced by itraconazole is 4%–8.6%, according to various studies [3]. A population-based survey from Spain enrolled 61858 patients to explore the risk of serious skin disorders among users of oral antifungal products [16]. Only 2 of 15329 itraconazole users developed serious, adverse skin reactions; 1 user developed angioedema, and the other, erythema multiforme. Other sporadic cases of cutaneous events associated with itraconazole include acute generalized exanthematic pustulosis, purpuric eruption, and infiltrative erythematous papules and macules [17].

According to an investigation by Tuccori [14], fatal hepatitis can develop after itraconazole treatment. At present, liver transplantation is used to treat patients with druginduced hepatic failure. Our review of the literature showed 3 deaths caused by itraconazole-associated hepatotoxicity [14]. Srebrnik reported the case of a patient with itraconazole-induced hepatic failure who survived after a successful liver transplantation [15]. Our results show the benefits of corticosteroids in the management of itraconazole-induced hepatic failure. In summary, monitoring of liver function during itraconazole use is important. Most of the hepatic injuries induced by itraconazole are self-limited, and patients generally recover after withdrawal of the offending drug. However, corticosteroid treatment should be used if the patient does not improve after a conservative treatment.
